# Meditative Movement Affects Working Memory Related to Neural Activity in Adolescents: A Randomized Controlled Trial

**DOI:** 10.3389/fpsyg.2020.00931

**Published:** 2020-05-12

**Authors:** Hojung Kang, Seung Chan An, Nah Ok Kim, Minkyu Sung, Yunjung Kang, Ul Soon Lee, Hyun-Jeong Yang

**Affiliations:** ^1^Korea Institute of Brain Science, Seoul, South Korea; ^2^Department of Brain Education, Global Cyber University, Cheonan-si, South Korea; ^3^Department of Integrative Biosciences, University of Brain Education, Cheonan-si, South Korea; ^4^Department of Integrative Health Care, University of Brain Education, Cheonan-si, South Korea

**Keywords:** meditative movement, working memory, EEG, exercise, executive function, cognitive function

## Abstract

Numerous studies have revealed that meditative movement changes brain activity and improves the cognitive function of adults. However, there is still insufficient data on whether meditative movement contributes to the cognitive function of adolescents whose brain is still under development. Therefore, this study aimed to uncover the effects of meditative movement on the cognitive performance and its relation with brain activity in adolescents. Forty healthy adolescent participants (mean age of 17∼18) were randomly allocated into two groups: meditative movement and control group. The meditative movement group was instructed to perform the meditative movement, twice a day for 9 min each, for a duration of 3 weeks. During the same time of the day, the control group was instructed to rest under the same condition. To measure changes in cognitive abilities, a dual n-back task was performed before and after the intervention and analyzed by repeated two-way analysis of variance (ANOVA). During the task, electroencephalogram signals were collected to find the relation of brain activity with working memory performance and was analyzed by regression analysis. A repeated two-way ANOVA with Bonferroni correction showed that working memory performance was significantly increased by meditative movement compared with the retest effect. Based on regression analysis, the amplitude of high-beta rhythm in the F3 channel showed a significant correlation with dual n-back score in the experimental group after the intervention, while there was no correlation in the control group. Our results suggest that meditative movement improves the performance of working memory, which is related to brain activity in adolescents.

**Clinical Trial Registration:** cris.nih.go.kr/cris, identifier KCT0004706.

## Introduction

Working memory is a subcomponent of executive function (Baddeley, 1992). Executive control is described as a subset of processes involved in the selection, scheduling, and coordination of computational processes underlying perception, memory, and action (Meyer and Kieras, 1997). Working memory consists of the real-time processing and retention of incoming information using temporary storage. It affects the shifting of information from short-term memory to long-term memory. Previous studies of working memory and other higher-order cognitive functions have found that these processes continue to mature during late adolescence (Brahmbhatt et al., 2008; Thomason et al., 2009; Andre et al., 2016), when they are critical for the successful maturation of one’s learning and memory into adulthood (Marosi et al., 1992; Ramscar and Gitcho, 2007).

In childhood and adolescence, the brain undergoes multifaceted and regionally differentiated maturational processes. Cognitive development in childhood and adolescence is achieved through a dynamic interaction between structural maturation and learning (Lebel and Beaulieu, 2011). In brain mapping research of normal children, brain growth is most prominent in frontal and occipital regions and changes in cortical thickness are highly significant in the dorsomedial prefrontal cortex (Sowell et al., 2004). A recent study also found that improvement in the working memory of children and adolescents were associated with cortical volume reduction in brain area, including bilateral prefrontal regions, but does not correlate with gender and age (Tamnes et al., 2013). In particular, frontotemporal connections have been shown to mature even after 20 years of age, and were reported to develop more slowly than any other region (Lebel et al., 2008). Significant clusters were identified in the white matter of the frontal and temporal lobes that are associated with working memory performance (Charlton et al., 2010). These results imply that improvements in the cognitive ability of children and adolescents accompany structural brain changes, especially in the prefrontal regions.

The characteristics of meditative movement, such as Qigong, Tai Chi, and yoga, include a conscious practice involving movement, a meditative state of mind, attention to breathing, and deep relaxation (Larkey et al., 2009). Previous studies suggest that meditative movement can change brain structure and improve adult cognitive ability (Wei et al., 2013; Brunner et al., 2017). Wei et al. (2013) compared and analyzed the brain structure between a long-term Tai Chi-practicing group (14 ± 8 years) and a control group and found that Tai Chi practice could thicken the cortical regions of the precentral gyrus, insula sulcus and middle frontal sulcus in the right hemisphere and the superior temporal gyrus, medial occipitotemporal sulcus, and lingual sulcus in the left hemisphere. Brunner et al. (2017) has revealed that yoga training was associated with significant improvement on both the maintenance and manipulation of working memory in young adults. Although these findings indicate the positive effects of meditative movement on brain structure and cognitive function in adults, it is not clear whether it is consistently applied to adolescents whose brain structural maturation is actively in progress. Recently, mindfulness meditation has been reported to enhance working memory performance in adolescents (Quach et al., 2016). This suggests that not only dynamic exercise (Tomporowski et al., 2008) but also static meditation improves the working memory of adolescents. Therefore it is speculated that meditative movement including meditative components of slow movement compared to regular exercise, may affect working memory and brain activity of adolescents.

The recording of electroencephalogram (EEG) activity used to study brain functionality has been extensively investigated during application of the n-back task. In addition, neural oscillations at specific frequencies have been shown to be related to specific cognitive processes. Theta power has been reported to play an important role in working memory (Sammer et al., 2007). In particular, theta activity has been reported to be associated with short-term memory tasks (Klimesch, 1996), maintenance (Sarnthein et al., 1998), and information retrieval (Klimesch et al., 2001). Furthermore, theta power involved in working memory has shown potential to be an EEG biomarker that can estimate an individual’s intelligence (Luo and Zhou, 2020). The role of alpha power has been associated with top-down functions (Von Stein et al., 2000; Palva and Palva, 2007; Sauseng et al., 2009a, b). However, in contrast to theta activity, alpha activity seems to reflect inhibitory activity (Romei et al., 2008; Sauseng et al., 2009a). Beta power increases as the working memory load increases (Deiber et al., 2007; Chen and Huang, 2016); however, increased working memory load has also been associated with beta desynchronization (Bočková et al., 2007; Krause et al., 2010). Beta power has been suggested to be associated with increased working memory performance due to the more effective filtering of irrelevant information (Zanto and Gazzaley, 2009). Therefore, the results of neural oscillation analyses can be used to estimate the changes in cognitive function as a result of meditation practice in our study.

In the current work, we hypothesized that meditative movement may increase the working memory performance of adolescents and the increase of working memory performance may correlate with increase of theta power, decrease of alpha power, and increase or decrease of beta power in adolescent brains. To this end, we randomly allocated 40 participants into either a meditative movement or control group. For the meditative movement group, participants were guided to practice meditative movement for 9 min per session, 2 sessions per day, for 3 weeks. For the control group, participants were guided to relax for the same time. Before and after the intervention, working memory performance was measured by scores of dual n-back task and EEG was measured to detect simultaneous brain activity. In the current research, meditative movement for adolescents improved working memory but not with other frequencies.

## Materials and Methods

### Participants

Healthy adolescent volunteers without any history of neurological or neuropsychiatric disorders, or hearing problems, participated in the research. The participants were recruited from the Benjamin School for Character Education, located in the South Korea. In order to see the effects of the intervention on working memory, the participants were randomly divided into two groups: the experimental and control groups (Table 1). They were all right-handed according to the Edinburgh Handedness

**TABLE 1:** Demographic characteristics of participants.

**Characteristics**	**Experimental group (*n* = 20)**	**Control group (*n* = 20)**	**Statistics**	
			***t* or x^2^**	***p***
**Gender, *n* (male/female)**	11/9	11/9	0	1
**Age, years (mean ± SD)**	17.55 ± 0.83	17.40 ± 0.68	0.0869	0.5344
**Edinburgh Handedness Inventory (mean ± SD)**	80.40 ± 14.87	76.33 ± 13.12	0.9175	0.3647

*Chi-squared test and *t*-tests were performed for gender and age/edinburgh handedness inventory data, respectively.*

**FIGURE 1:**
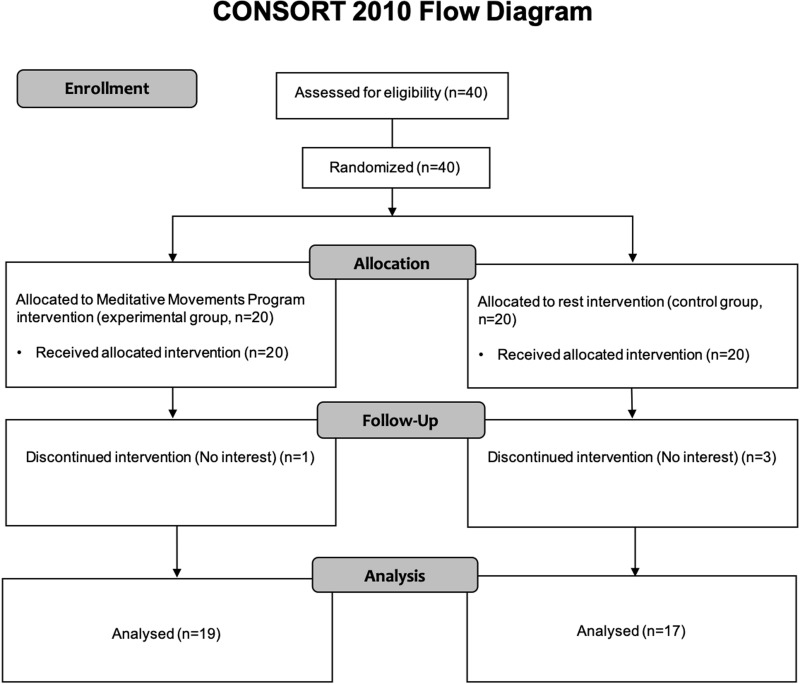
CONSORT 2010 flow diagram. Forty participants were recruited. The participants were randomly divided into two groups: experimental and control group. Thirty-six participants (Experimental, 19; Control, 17) completed the study, with 4 dropouts (Experimental, 1; Control, 3). The dropouts said that they do not have interests.

Inventory (Oldfield, 1971) and had had normal or “corrected to normal” vision. Volunteers who have experiences in any mind–body trainings such as yoga, meditation, Qigong and Tai Chi, were excluded. Among 40 recruited participants, 36 individuals were analyzed: one and three participants in the experimental and control group, respectively, did not complete the study because of lack of interest (Figure 1). The Institutional Review Board of the University of Brain Education approved this study. Written informed consent/assent was obtained from all children and their parents. The current protocol is registered as a clinical trial in the Clinical Research Information Service (CRIS registration number: KCT0004706).

### Meditative Movement Training

In this study, an instructor with extensive training in DahnMuDo guided the experimental groups. DahnMuDo is a comprehensive system of meditative movement that is derived from Korean healing and martial arts tradition (Dahn Yoga Education, 2006). The training program used in the present study was composed of three body positioning exercises with eyes closed. The first exercise (Single Leg Stance: Dong-Nip-Bo) involved extension of the right arm 90° to the front and holding the left foot to maintain the posture. The second action reversed the first exercise along with the direction. The last exercise (Horse Stance: Ki-Ma-Bo) involved bending the knees similar to sitting on a chair after spreading legs to shoulder width, and maintaining the posture. The training program is a part of an after-school class program in Korea for the healthy development of the mind and body of students. Participants were asked to perform each step for 3 min. Participants in the experimental group were instructed to perform the training program twice a day, for 9 min each time, for 3 weeks. Simultaneously, subjects were asked to keep a clear or calm state of mind while regulating their breathing. Participants in the control group were seated and asked to relax during that time. All tasks for both groups were performed in the same space under the guidance of the instructor.

### Dual n-Back Task

**FIGURE 2:**
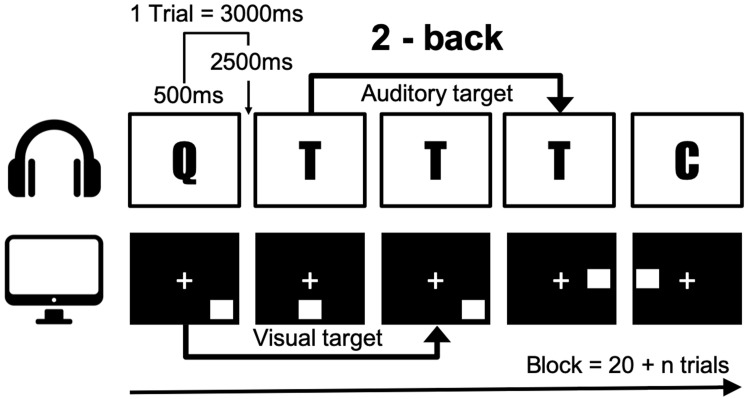
The n-back task illustrated for a 2-back condition. A letter was presented as an auditory stimuli simultaneously when a spatial material was presented visually.

In order to measure working memory, we conducted a dual n-back task that utilizes audio-visual stimuli (Jaeggi et al., 2008). In this task, the square marker was displayed sequentially in eight locations, and one of the eight syllables was heard simultaneously through earphones (Figure 2). Participants responded manually to stimuli according to n-back conditions by pressing the letter “A” on a standard keyboard for visual targets, and the letter “L” for auditory targets. No responses were required for non-targets. Accuracy was measured depending on the exact number of responses. A wrong response to the non-target was penalized by score reduction. After each block, the individual performance was analyzed, and in the following block, the level of n was adapted accordingly. If the accuracy was higher than 80%, the level of n was increased by 1, and it was decreased by 1 if the accuracy was less than 60% and the first step was lowered. In all other cases, the level of n was maintained. A single block comprised 20 + n trials and one session consisted of 6 blocks amounting to time of approximately 25 min. A total of 6 blocks were conducted starting with level 2. The total degree of difficulty was measured up to 1–4 levels. Working memory performance was estimated as the average of the whole dual n-back level, except for the first block. In this study, participants performed an adequate number of exercises enough to understand dual n-back with level 2 during the pre-interview, specifically till the number of errors reaches less than 5. Before the intervention, the participants performed the dual n-back task. Three weeks later, they performed the same task to quantify the effects of meditative movement. All participants were prohibited from dual n-back task training during the 3-week study period.

### EEG Data Acquisition

The Emotiv EPOC wireless headset (14 channels^1^, United States) was used to record brain activity during the dual n-back task. The EEG data were collected via 14 electrodes located at AF3, F7, F3, FC5, T7, P7, O1, O2, P8, T8, FC6, F4, F8, and AF4 based on the American EEG Society Standard with two references (i.e., CMS/DRL). The sampling rate was 256 Hz, continuous EEG were digitally band pass filtered at 0.3–45 Hz and the digital notch filters at 60 Hz.

### EEG Data Analysis

EEG data were segmented into epochs from −500 to 2,500 ms time-locked to stimulus onset. The segmented EEG data in the first block were excluded to preserve the statistical power of working memory performance. The EEG data epochs were submitted to extended infomax ICA (Lee et al., 1999) using runica (Makeig et al., 1997) from the EEGLAB toolbox to eliminate eye-blink or eye-movement artifacts. The activated components from each subject were first assessed and categorized as brain activity or non-brain artifact (e.g., muscle, line noise, or eye-movement activity) by visual inspection. Pre-processed EEG data were used for fast Fourier transformation to estimate the power of each brain rhythm including theta (4–8 Hz), alpha (8–13 Hz), low-beta (13–30 Hz), and high-beta (30–40 Hz) during the working memory task via a fieldtrip toolbox (Oostenveld et al., 2011) with Matlab (MathWorks). The EEG power was estimated from the whole epochs (i.e., −500 to 2500 ms). Individual band power (i.e., theta, alpha, low-beta, and high-beta) was averaged across epochs.

### Statistical Analysis

Working memory scores were measured using the average of the level performed in the 2–6 block except the first block to preserve statistical power. To examine the changes, working memory scores were analyzed by repeated measures analysis of variance (ANOVA). Multiple comparisons problem was corrected using critical values from the *t* distribution, after a Bonferroni adjustment. A regression analysis of individual band power and working memory scores was conducted to evaluate the changes in neural activity associated with working memory tasks, for each of the 14 channels. A least squares regression was used to determine the regression coefficients. All statistical analyses were performed using Matlab (MathWorks).

## Results

### Comparison of Working Memory Performance Between Experimental and Control Group

**FIGURE 3:**
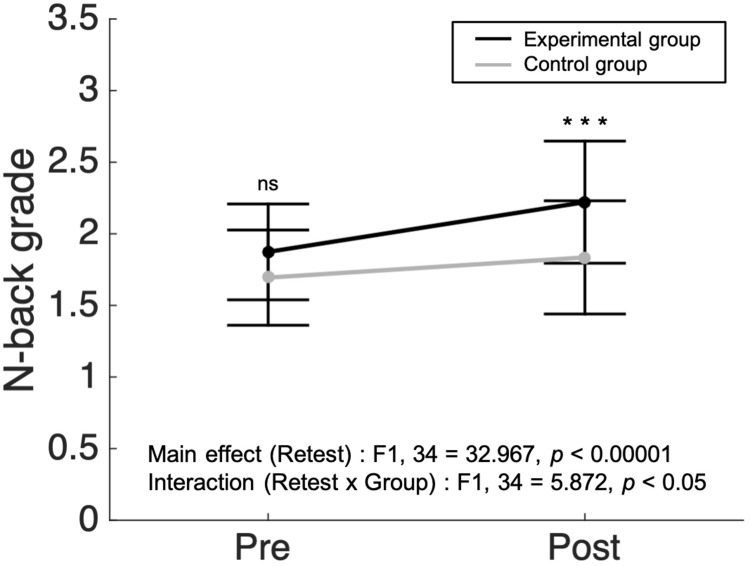
A larger increase in n-back grade in meditative movement group compared to control group. The dots and error bars of pre- and post-intervention indicate average ± standard deviation of N-back grade. In two-way repeated measured analysis of variance, significant results were found in the main effect of retest (*F*_1,34_ = 32.967, *p* < 0.00001) and the interaction between retest and group (*F*_1,34_ = 5.872, *p* < 0.05). In *post hoc* analysis, the post-experimental group showed a significant increase in working memory score, compared to the post-control group (Bonferroni correction, ***p < 0.001). ns, not significant.

There were no significant differences in working memory scores between the experimental (*N* = 19) and control group (*N* = 17) before the intervention (*p* = 0.116, two sample *t*-test). After the 3 week intervention period, measures of working memory performance were analyzed via a two-way repeated measures ANOVA with group (experimental/control) and retest (Pre/Post) as factors. We found a main effect in the retest factor (*F*_1,34_ = 32.967, *p* < 0.00001, $\eta_{\text{p}}^{2}$ = 0.492, Figure 3). Both groups showed improvement in working memory performance after the intervention. In the interaction analysis between group and retest factors, a significant interaction was found (*F*_1,34_ = 5.872, *p* < 0.05, $\eta_{\text{p}}^{2}$ = 0.147, Figure 3). *Post hoc* tests using the Bonferroni correction revealed that there was no significant difference between the experimental group and the control group before the intervention in the working memory score (1.87 ± 0.33 vs. 1.69 ± 0.33, respectively). However, the averaged score of the experimental group (2.22 ± 0.43) was substantially superior compared to that of the control group (1.84 ± 0.40) after the intervention (*p* < 0.001, with Bonferroni *post hoc* test). The findings suggest that meditative movement training improves working memory regardless of retest effects.

### Correlation of EEG Channel With Working Memory

**FIGURE 4:**
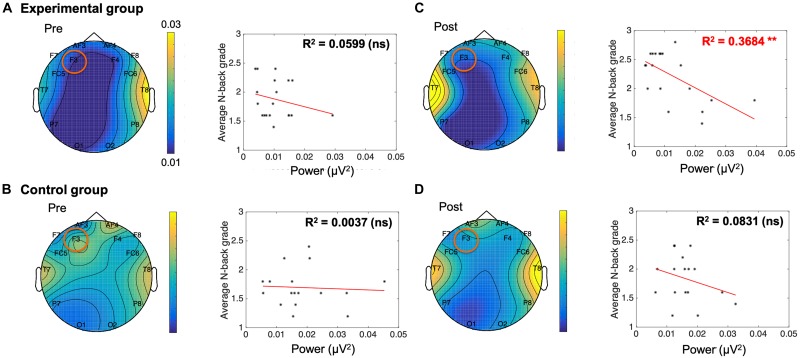
A negative correlation between electroencephalogram beta power of F3 and n-back grade in the post-meditative movement group. **(A,B,D)** The conditions without training showed no significant correlation between n-back grade and beta power. **(C)** The beta power of F3 was negatively correlated with working memory performance in participants exposed to meditative movement for 3 weeks (*R*^2^ = 0.368, **p < 0.01). ns, not significant. *p < 0.05, ***p < 0.001.

Individual band power (i.e., theta, alpha, low-beta, and high-beta) represents the level of neural activation according to the individual levels of working memory, which is applied equally across the 14 channels. The results were used as regressors to calculate the regression coefficient with the level of dual n-back task under each condition: the pre-experimental, post-experimental, pre-control, and post-control groups. We performed regression analysis between working memory score and band power of theta (Table 2), alpha (Table 3), low-beta (Table 4), and high-beta (Table 5) under each condition. For theta, alpha and low-beta band power, no channels were found to correlate with working memory. However, at the F3 site of high-beta band power, there was a tightly coupled neuronal activity with working memory score in the post-experimental group. Representative EEG recordings showing high-beta band power for each condition are given in Figure 4. In the pre-training phase, neither group showed correlation between high-beta power and working memory performance at F3 (Figures 4A,B). These results indicate that in both groups, no high-beta power affecting working memory performance was observed before the intervention period. At the site of the F3 channels corresponding to the dorsolateral prefrontal cortex (DFC), a significant correlation was found between averaged high-beta power and averaged working memory score in the post-experimental group (*R*^2^ = 0.368, *p* < 0.01, Figure 4C), but not in the post-control group (Figure 4D). These results suggest that through meditation movement training, brain activity that relates with working memory performance was increased in the DFC where the F3 channel is located.

**TABLE 2:** Regression analysis for working memory score in theta rhythm.

**Theta**	**Pre-experimental group**		**Post-experimental group**		**Pre-control group**		**Post-control group**	
				
**Channels**	***R*^2^**	***p***	**R^2^**	***p***	***R*^2^**	***p***	***R*^2^**	***p***
AF3	0.0243	0.5243	0.0319	0.4646	0.0355	0.4687	0.0441	0.4187
F7	0.0171	0.594	0.0263	0.5069	0.021	0.5786	0.0016	0.879
F3	0.1346	0.1223	0.0657	0.2895	0.0474	0.4014	0.001	0.9034
FC5	0.017	0.5949	0.0004	0.9367	0.0036	0.8185	0.0224	0.5664
T7	0	0.9836	0.1425	0.1111	0.0037	0.816	0.0496	0.3901
P7	0.0004	0.9317	0.0417	0.4014	0.0126	0.6678	0.0096	0.7089
O1	0.0605	0.31	0.064	0.2961	0.1021	0.2111	0.0025	0.8482
O2	0.0585	0.3184	0.0017	0.8664	0.0976	0.2221	0.0018	0.8726
P8	0.0237	0.5288	0.0815	0.2362	0.0222	0.5684	0.029	0.5138
T8	0.1988	0.0557	0.0767	0.2511	0.0037	0.8157	0.0175	0.613
FC6	0.06	0.3121	0.0018	0.8643	0.0022	0.8579	0.1296	0.1558
F4	0.2114	0.0476	0.1158	0.1539	0.0573	0.3548	0.022	0.5699
F8	0.1223	0.1422	0.0641	0.2955	0.0043	0.8018	0.002	0.8634
AF4	0.0168	0.5972	0.0259	0.5104	0.0864	0.2521	0.0131	0.6617

**TABLE 3:** Regression analysis for working memory score in alpha rhythm.

**Alpha**	**Pre-experimental group**		**Post-experimental group**		**Pre-control group**		**Post-control group**	
				
**Channels**	***R*^2^**	***p***	***R*^2^**	***p***	***R*^2^**	***p***	***R*^2^**	***p***
AF3	0	0.9895	0.076	0.2533	0.0001	0.9702	0.0319	0.4925
F7	0.0066	0.74	0.0365	0.4336	0.0069	0.7516	0.0262	0.5347
F3	0.0637	0.297	0.1377	0.1178	0.05	0.3884	0.0304	0.5035
FC5	0.0019	0.8596	0.0023	0.8452	0.0007	0.919	0.006	0.7683
T7	0.0035	0.81	0.1151	0.1553	0	0.9845	0.0016	0.8783
P7	0.0169	0.5963	0.0305	0.4745	0.028	0.521	0.0445	0.4161
O1	0.1015	0.1836	0.0081	0.7145	0.0435	0.4216	0.0068	0.7535
O2	0.0154	0.6123	0.0032	0.8174	0.0103	0.6983	0.1458	0.1304
P8	0.0859	0.2234	0.003	0.8245	0.0024	0.8518	0.1678	0.1025
T8	0.1002	0.1867	0.0219	0.545	0.0003	0.9517	0.0001	0.9687
FC6	0.0306	0.4737	0	0.9875	0.0079	0.7338	0.144	0.133
F4	0.1646	0.0848	0.1325	0.1255	0.0005	0.9298	0.0351	0.4714
F8	0.0972	0.1937	0.0176	0.5883	0.002	0.8641	0.0138	0.6533
AF4	0.004	0.7964	0.0371	0.4294	0.0095	0.7103	0.0023	0.8545

**TABLE 4:** Regression analysis for working memory score in low-beta rhythm.

**L-beta**	**Pre-experimental group**		**Post-experimental group**		**Pre-control group**		**Post-control group**	
				
**Channels**	***R*^2^**	***p***	***R*^2^**	***p***	***R*^2^**	***p***	***R*^2^**	***p***
AF3	0.0668	0.2852	0.2166	0.0446	0	0.9881	0.0062	0.7635
F7	0.0404	0.4096	0.0004	0.9367	0.0243	0.5502	0.0624	0.3336
F3	0.1097	0.166	0.2746	0.0213	0.0145	0.6452	0.0862	0.2527
FC5	0.0005	0.9305	0.0111	0.6672	0.0041	0.8073	0.0131	0.6614
T7	0.0026	0.8343	0.1079	0.1697	0.0389	0.4479	0.1034	0.2083
P7	0.0113	0.6651	0.0001	0.9635	0.0287	0.5156	0.0004	0.9375
O1	0.0932	0.2037	0.0123	0.651	0.0341	0.4783	0.0239	0.5536
O2	0.0582	0.3196	0.0195	0.5685	0.0873	0.2496	0.129	0.1569
P8	0.032	0.464	0.0173	0.5915	0.0158	0.6311	0.0765	0.2824
T8	0.0839	0.2291	0.0343	0.4479	0.0296	0.5094	0.0813	0.2673
FC6	0.0328	0.4584	0.0393	0.4157	0.0021	0.8609	0.0466	0.4053
F4	0.0147	0.6213	0.0264	0.506	0.0173	0.6152	0.0258	0.538
F8	0.1076	0.1704	0.092	0.2068	0.0606	0.341	0.005	0.7877
AF4	0.0123	0.6511	0.0068	0.7363	0.0111	0.6873	0.0046	0.7958

**TABLE 5:** Regression analysis for working memory score in high-beta rhythm.

**High-beta**	**Pre-experimental group**		**Post-experimental group**		**Pre-control group**		**Post-control group**	
				
**Channels**	***R*^2^**	***p***	***R*^2^**	***p***	***R*^2^**	***p***	***R*^2^**	***p***
AF3	0.0875	0.2189	0.2822	0.0193	0.0088	0.7203	0	0.9901
F7	0.0321	0.4632	0.0622	0.3031	0.0902	0.2414	0.0515	0.3811
**F3**	0.0599	0.3126	**0.3684**	**0.0059**	0.0037	0.8165	0.0831	0.2618
FC5	0.0001	0.9649	0.0367	0.4318	0.0334	0.4827	0.0081	0.7305
T7	0.0022	0.8499	0.0641	0.2957	0.0029	0.8363	0.0637	0.3282
P7	0.0078	0.7184	0.0177	0.5876	0.0173	0.6146	0.0001	0.9774
O1	0.0506	0.3544	0.0183	0.5808	0.0135	0.6576	0.0052	0.7834
O2	0.0819	0.235	0.0228	0.537	0.1718	0.0981	0.0884	0.2466
P8	0.0022	0.8479	0.0183	0.5805	0.0518	0.3796	0.0644	0.3259
T8	0.0977	0.1925	0.0805	0.2392	0.035	0.4723	0.077	0.281
FC6	0.0408	0.4067	0.0713	0.2692	0.0015	0.8834	0.0096	0.7078
F4	0.0021	0.8514	0.0002	0.9603	0.0881	0.2474	0.0106	0.6945
F8	0.0735	0.2615	0.1925	0.0602	0.1745	0.0952	0.0105	0.696
AF4	0.0011	0.8918	0.006	0.7523	0.0047	0.7943	0.0192	0.5963

*A significant effect (*p* < 0.01) is indicated by bold characters.*

## Discussion

We designed an experiment to determine the effects of a 3-week meditative movement training on the working memory and its relation with neural activity of adolescent brains. In this study, we found that the meditative movement group showed a significant increase in dual n-back accuracy when compared with the control group at post-intervention. This suggests that the experimental group which underwent meditative movement training for 3 weeks had improved working memory ability compared to the control group. The n-back task requires not only the storage and continual updating of information in working memory, but also interference resolution. The n-back task continuously displays stimuli between intervals of several seconds. The participants must determine whether the current stimulus matches what was indicated before n trials, where n is the number of variables that can be adjusted up or down to increase or decrease the cognitive load, respectively. Therefore, meditative movement seems to have a positive effect on memory capacity, judgment ability, or stress management based on intelligent load. Interestingly, the relationship between working memory performance and the degree of high beta rhythm at F3 was observed by regression analysis based on dual n-back accuracy and power of brain rhythms derived from EEG. After training, the beta rhythm of F3 from the prefrontal cortex showed a negative correlation with an accurate response rate. Neural activity associated with beta power has been reported to be involved in the efficient filtering of irrelevant information in the processing of working memory (Zanto and Gazzaley, 2009). A deficit in inhibiting the processing of irrelevant information has been suggested as a cause of a broad spectrum of cognitive deficits (Hasher and Zacks, 1988). Therefore, improvement in working memory performance by meditative movement training in our study might be induced by the enhanced filtering of irrelevant information, which is related with beta power, but this needs further investigation.

The prefrontal cortex is thought to be the most important brain region in terms of working memory based on extensive studies in monkeys, which demonstrated that lesions associated with the prefrontal cortex impaired performance on delayed response tasks (Baddeley, 1998, 2000; Petrides, 2000). Lesions involving the DFC decreased the performance of working memory, but not short-term memory, in monkeys (Petrides and Milner, 1982). These results indicate that the DFC is more likely involved in information processing and executive function rather than information retention. Human studies showed that the DFC plays an important role in the neuropsychological performance of patients with focal neurological lesions (Owen et al., 1990; Owen et al., 1996), and exposure to transcranial magnetic stimulation on the F3 location provides benefits to working memory (Fregni et al., 2005; Preston et al., 2010). In addition, previous EEG studies have reported that gamma oscillation and GABA levels in the F3 channel may play an important role of working memory performance in schizophrenia (Chen et al., 2014). These results suggest that the left DFC, where F3 is located, is an important brain region for working memory. Consistent with previous reports on the function of this location, we observed the changes of beta rhythm correlated with working memory specifically at this location in the current study.

As participants of meditative movement keep focusing on their body sensations during training, meditative movement can be said to include two training components: mental and physical component. In relation to the mental component, meditative movement shows effects on maintenance of a relaxed and attentive mind (Tei et al., 2006, 2009). Compared to a normal healthy condition, under chronic stress conditions, the neuronal activity of the prefrontal cortex is significantly diminished (Arnsten, 2009). Stress reduces the neuronal activity related with working memory in the prefrontal cortex (Qin et al., 2009; Mika et al., 2012). Meditative movement induces stress-alleviating effects (Posadzki et al., 2010; Wang et al., 2014) and activates the prefrontal cortex (Cheng et al., 2010). Not only the mental component, but also the physical component of meditative movement may affect working memory. Physical exercise was shown to improve working memory (Baddeley, 1992; Aben et al., 2012), which is critical for a wide range of cognitive functions (Daneman and Tardif, 1987; Engle et al., 1990; Kail, 2007). The exercise-cognition relationship was established by the increase in academic performance following exercise in school-aged children (Dwyer et al., 2001; Tomporowski et al., 2008). In this study, our findings of improvement in working memory performance and its relationship with neural activity in the prefrontal cortex by meditative movement are consistent with previous reports mentioned above.

Meditative movement improved working memory performance, which is related to neural activity of the prefrontal cortex in our results. Working memory plays important roles in cognitive function (Daneman and Tardif, 1987; Engle et al., 1990; Kail, 2007). During adolescence, cognitive development progresses actively, which is made up of dynamic interaction between brain structural maturation and learning (Lebel and Beaulieu, 2011). Improvement in the working memory of adolescents is related with brain structural changes including the bilateral prefrontal regions (Tamnes et al., 2013). Therefore, changes in the neural activity of the prefrontal cortex and improvement in working memory by meditative movement suggests that it can be utilized as a learning tool which induces positive interaction for brain maturation during adolescence.

According to the recent meta regression analysis, coordinative exercise was most effective for improving cognitive function (Ludyga et al., 2020). In view of cognition, the exercise modes are divided into physical training whose character is repetitive and automatic, and motor training in which coordinative exercise is included. While physical training indirectly affects cognition through improvements in cardiovascular strength by training intensity, motor training directly affects cognition through neuromuscular activation (Netz, 2019). This implies underlying mechanisms how meditative movement, which can be categorized into motor training, improved working memory performance in the current study. Our observations suggest that not only traditional physical training but also meditative movement which demands one’s focus although the motion speed is relatively slow can be used as an effective tool for improving cognition. Especially, this is practically meaningful because the meditative movement can be used to improve cognition of people under various physical condition.

In this study, we observed that the meditative movement significantly increased working memory score and affected the neuronal activity associated with working memory in adolescents. In regression analysis, the coefficient of determination (*R*^2^ = 0.368) between EEG F3 channels and working memory performance showed a rather weak correlation. For social science data, the effect size of 0.25 is suggested to interpret as moderate effect (Ferguson, 2009). According to the criteria, our effect size of 0.368 corresponds to the moderate effect. In order to guarantee the repeatability of the findings, more attention should have been paid to increase statistical power by strategies, which is included to the limitations of the current study. Recently, a meta-analysis study has presented results implying sex-specific recommendations on how the effects of exercise on cognition in healthy individuals can be optimized by potential moderators such as exercise intensity, type, and session duration (Ludyga et al., 2020). In the current study, the percentage of female participants is 45%, which may dilute the overall effect of the exercise intervention due to the mixed use of gender, which can be another limitation of the study. For further researches, experimental design should be performed under consideration of gender difference. Moreover, Ludyga et al. (2020) revealed that the effect size increased with expending the session duration, lasting the exercise intervention over 20 weeks, and increasing the sample size, which are included to the limitations of the current study. In addition, we controlled several confounding factors such as age, handedness, and vision, which might affect brain activities. However, other confounding factors including factors of affective and motor aspects were not strictly controlled, which is a limitation of the study. The current results suggest the connectivity between meditative movement and cognitive function and are meaningful in following three aspects. First, this study contributes to accumulation of knowledge about the effects of meditative movement during adolescence, when there has been insufficient data of mind–body training for adolescence. Second, it provides scientific evidence for the application of meditation training to actual education. Especially, our results suggest that not only dynamic exercise (Tomporowski et al., 2008) as previously known, but also meditation training accompanied by slow movements with high concentration can be a good tool for improvement of working memory, which is important for cognitive function (Daneman and Tardif, 1987; Engle et al., 1990; Kail, 2007), in adolescents. This presents a broad application possibility for cognitive development tools to adolescent students with various physical conditions. Third, it presents an evidence-based direction for further study.

Future studies include the investigation of the mechanisms which may cause the changes observed here. Meditative movement may improve cognitive abilities through mechanisms involving stress management or physical fitness. To understand this, it is necessary to study the relationship between stress responsiveness, physical changes, and cognitive ability after meditative movement training. In addition, changes in brain activation during meditative movement are also expected to provide very important clues. One further interesting question is what common brain activity patterns and networks are shown during meditation movement and working memory, and how this affects cognitive performance. Another interesting question in the next study is whether there is a link between brain activation, stress, and physical changes during meditative movement training, and whether there is a change in brain area activity associated with the maturation of working memory. The cognitive improvement of adolescents who performed meditative movement training in the current study warrants its application potential to education and further researches on meditative movement training, as a mean to enhance adolescent brain development.

## Data Availability Statement

All datasets generated for this study are included in the article/supplementary material.

## Ethics Statement

The studies involving human participants were reviewed and approved by the Institutional Review Board of the University of Brain Education. Written informed consent to participate in this study was provided by the participants’ legal guardian/next of kin.

## Author Contributions

The authors contributed to the work as described in the following. HK, SA, and H-JY: conception and design of the work and drafting the work. HK, NK, UL, and YK: conducting the experiment and collecting the data. HK, MS, and H-JY: analysis and interpretation of data for the work. All authors reviewed the manuscript and approved the final version for the publication.

## Conflict of Interest

The authors declare that the research was conducted in the absence of any commercial or financial relationships that could be construed as a potential conflict of interest.

## Footnotes

1 www.emotiv.com
